# Defining the Differential Corticosteroid Response Basis from Multiple Omics Approaches

**DOI:** 10.3390/ijms252413611

**Published:** 2024-12-19

**Authors:** Melody Ramirez-Falcon, Eva Suarez-Pajes, Carlos Flores

**Affiliations:** 1 Research Unit, Hospital Universitario Ntra. Sra. de Candelaria, Instituto de Investigación Sanitaria de Canarias, 38010 Santa Cruz de Tenerife, Spain; 2 Centro de Investigación Biomédica en Red de Enfermedades Respiratorias (CIBERES), Instituto de Salud Carlos III, 28029 Madrid, Spain; 3 Genomics Division, Instituto Tecnológico y de Energías Renovables, Granadilla de Abona, 38600 Santa Cruz de Tenerife, Spain; 4 Facultad de Ciencias de la Salud, Universidad Fernando Pessoa Canarias, 35450 Las Palmas de Gran Canaria, Spain

**Keywords:** corticosteroids, glucocorticoids, genomics, transcriptomics, epigenomics, proteomics, metabolomics

## Abstract

Since their discovery, corticosteroids have been widely used in the treatment of several diseases, including asthma, acute lymphoblastic leukemia, chronic obstructive pulmonary disease, and many other conditions. However, it has been noted that some patients develop undesired side effects or even fail to respond to treatment. The reasons behind this have not yet been fully elucidated. This poses a significant challenge to effective treatment that needs to be addressed urgently. Recent genomic, transcriptomic, and other omics-based approximations have begun to shed light into the genetic factors influencing interindividual variability in corticosteroid efficacy and its side effects. Here, we comprehensively revise the recent literature on corticosteroid response in various critical and chronic diseases, with a focus on omics approaches, and highlight existing knowledge gaps where further investigation is urgently needed.

## 1. Introduction

Corticosteroids (CSs) have been widely used in clinical care for over 70 years to treat inflammatory and autoimmune diseases due to their potent anti-inflammatory and immunosuppressive effects [1,2,3]. They are used in a broad range of chronic and acute inflammatory and autoimmune conditions such as acute respiratory distress syndrome (ARDS), chronic obstructive pulmonary disease (COPD), community-acquired pneumonia, asthma, septic shock, and, most recently, the coronavirus disease 2019 (COVID-19), among others.

Despite their demonstrated therapeutic benefits, CSs are associated with substantial adverse effects. Side effects include hyperglycemia, tachycardia, glaucoma, agitation, insomnia, anxiety, weight gain, hirsutism, immunosuppression, gastrointestinal bleeding, fluid retention, and muscle weakness, among others [4,5]. In addition, some patients do not respond to CS treatment or even develop worsening clinical outcomes, making their use controversial [6,7].

For that reason, the clinical guidelines of CS use are continually updated to align with the latest evidence on their administration recommendations. These guidelines provide insights into which patient populations are likely to benefit from CS therapy and define optimal dosing and treatment schedules. However, current guidelines are primarily based on clinical evidence and do not yet address the variability in patient responses to CS therapy. This gap underscores the need for a deeper understanding of the molecular factors influencing the individual responses to CS treatment.

Host genetic factors have been shown to play a central role in determining drug response, reinforcing the relevance of pharmacogenomics in clinical decision making [8,9]. Here, we aimed to comprehensively revise the recent literature leveraging omics approaches to identify molecular determinants of the CS response in different critical and chronic diseases. The integration of genetic and molecular data into the understanding of CS mechanisms of action will be essential for the advancement of precision medicine by shaping new guidelines and the clinical practice. Before moving on to the main topic, we will also introduce the mechanisms by which CSs exert their functions to expose the complexity and diversity of the physiological and immunological responses regulated by their activity.

## 2. Mechanisms of Glucocorticoid Action

CSs are a family of steroid hormones that include glucocorticoids (GCs) and mineralocorticoids (MCs). GCs, such as cortisol, cortisone, and corticosterone, regulate inflammation and immune responses, while MCs, like aldosterone, regulate sodium and water balance. Cortisol is the main endogenous GC in humans, and it is synthesized from cholesterol in the adrenal cortex. Its production is regulated by the hypothalamic–pituitary–adrenal (HPA) axis, following the circadian rhythm, peaking in the morning (10–15 μg/mL, 6:00–8:00 a.m.), and decreasing throughout the day [5]. Synthetic GCs have been used in clinical practice since their discovery in 1948 [10] to treat inflammation and autoimmune conditions, and they can be administered orally, intravenously, intramuscularly, topically, or via inhalation, each route offering different bioavailability and suitability depending on the condition treated.

### 2.1. Genomic Mechanisms of Glucocorticoid Action

**Figure 1:**
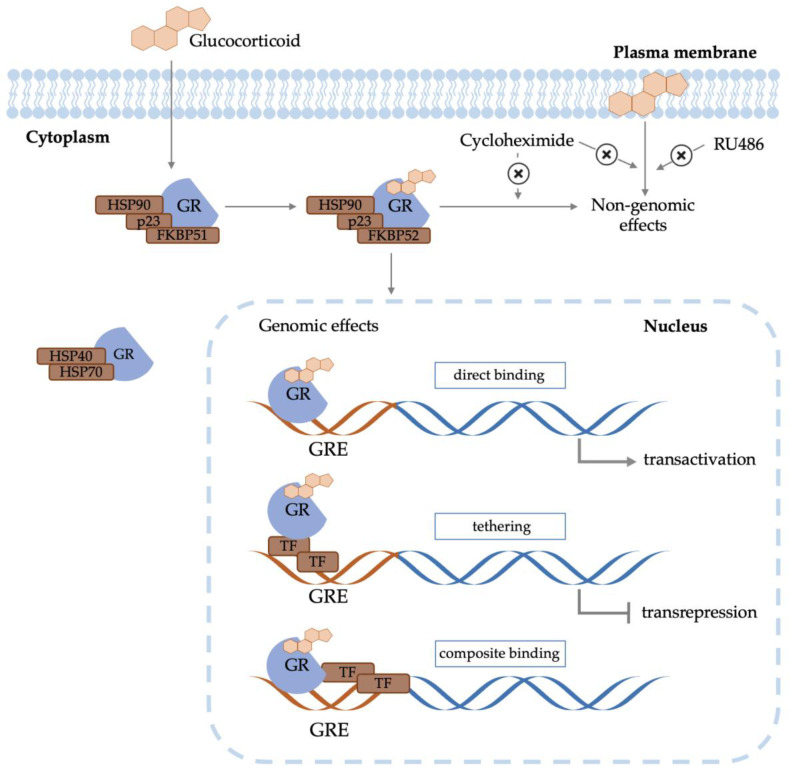
Schematic diagram of the genomic and non-genomic mediated glucocorticoid effects. GR: glucocorticoid receptor; GRE: glucocorticoid response element; and TF: transcription factor.

Once administered, GCs diffuse from the bloodstream to the cellular cytoplasm and bind to glucocorticoid receptors (GRs) (Figure 1). Before the binding, inactive GR monomers are located in the cellular cytoplasm bound to an HSP40/HSP70 chaperone complex, which facilitates the recruitment of the HSP90 protein [11,12,13]. The binding of HSP90 causes the release of the HSP40/HSP70 complex and the binding of p23 [14] and FKBP51, thus forming a mature GR–chaperone complex with a high GR ligand affinity [15]. After GC binding, the GR–chaperone complex substitutes FKBP51 for FKBP52 and undergoes a conformational change, forming a GC receptor–chaperone complex. The complex then translocates to the cell nucleus, where it dimerizes and binds to GC response elements (GREs) to regulate the transcription of inflammatory and immunological genes in three primary ways: by direct binding to accessible DNA, by tethering when it binds a transcription factor that in turn binds DNA, and by composite binding both to DNA and a DNA-bound transcription factor [16]. GCs can exert their function by suppressing transcription of inflammatory genes (also known as transrepression) or by stimulating the transcription of anti-inflammatory genes (also known as transactivation) [17].

### 2.2. Non-Genomic Mechanisms of Glucocorticoid Action

The onset of effects of the systemic GC activity usually occurs 3 to 8 h from the administration. This may be due to the intracellular GR binding and translocation to the nucleus. However, it has been shown that GCs can sometimes induce their effects within minutes. These rapid responses are mediated via non-genomic mechanisms, which can be exerted through interaction with cell membranes, with cytosolic GRs, or through the interaction with membrane-bound GRs [18]. Non-genomic effects are not mitigated by inhibitors of transcription (actinomycin D) or translation (cycloheximide), and when the effects are mediated through interactions with the cell membrane, they are not dampened by the GR antagonist RU486 either [18,19].

#### 2.2.1. Cell Membrane Mediated Effects

One of the non-genomic effects of GCs involves the modulation of basal intracellular calcium ([Ca^2+^]i) levels and agonist-induced calcium mobilization. It is believed that these effects are independent of GR activity, as they are unaffected by RU486. Therefore, GCs may intercalate at the cell membrane and influence ion transport. However, these effects on Ca^2+^ appear to be cell-type dependent [20,21,22], and the non-genomic nature of the effects on Ca^2+^ levels in immune cells has not been yet conclusively proven [23,24].

Furthermore, GCs reduce ATP production through interaction with cellular and mitochondrial membranes by the inhibition of oxidative phosphorylation and mitochondrial increased proton leak [17]. ATP is essential to immune cells for cytokine synthesis, migration, phagocytosis, and antigen processing and presentation. Therefore, its reduction contributes to the immunosuppressive effects of GCs.

GCs have also proven to inhibit N-formyl-methionyl-leucyl-phenylalanine-induced degranulation in human neutrophils in vitro, thus exerting an anti-inflammatory effect [25]. This effect was not prevented by RU486 or cycloheximide, also supporting the contribution of a GR-independent and non-genomic mediated process.

#### 2.2.2. GR-Mediated Effects

GCs have demonstrated to affect nitric oxide synthase (NOS) activity through GR-mediated non-genomic mechanisms [26]. This enzyme is responsible for the synthesis of nitric oxide (NO), which is involved in acute and chronic allergen-induced airway hyperresponsiveness (high levels of NO have been associated with epithelial damage, inflammation, and mucus hypersecretion).

Furthermore, GCs are suggested to influence other inflammatory mediators by non-genomic mechanisms of action. For instance, in adenocarcinoma cells, acute exposure to dexamethasone inhibited epidermal growth factor (EGF)-induced arachidonic acid release, a key inflammation mediator, through a GR-dependent (RU486-sensitive) and transcription-independent (actinomycin D-insensitive) mechanism [27].

In studies with primary CD4+ T cells derived from human peripheral blood mononuclear cells and Jurkat cell cultures, the dexamethasone pretreatment inhibited T cell receptor (TCR) signaling pathways, decreasing overall TCR signaling and activation [28]. This inhibitory effect of dexamethasone was reversed by RU486 treatment, indicating that it is likely a GR-dependent process.

Finally, another non-genomic mechanism of GC action involves binding of the GC receptor–chaperone complex to cytoplasmic mRNA, which induces its degradation and causes the downregulation of translations of proinflammatory transcripts [29,30,31].

Overall, the GC mechanisms of action exposed (Figure 1) show only a small portion of the broad processes through which GCs exert their functions. Furthermore, the risks and extent of the side effects are related to dose, the duration of the treatment, specific GCs administered, the time of daily administration, and the route of administration [5]. At the molecular level, lipophilicity and binding affinity of the chosen GC, and cell type also influence the treatment effects [32,33]. Given their extensive genomic, non-genomic, and cell type-dependent effects, and despite the ever-growing understanding of their mechanisms of action, there is still a need for further in-depth research to fully unravel the molecular mechanisms involved and broader implications.

## 3. Genomics of the Glucocorticoid Response

### 3.1. The Glucocorticoid Receptor and Candidate Gene Studies

The human GR (hGR) is a modular protein consisting of an N-terminal transactivation domain (NTD), a central DNA-binding domain (DBD), a C-terminal ligand-binding domain (LBD), and a flexible hinge region that separates the DBD from the LBD [34,35]. The hGR is the product of the *NR3C1* gene, which is located on chromosome 5q31.32 and contains nine exons [34,35]. Exons 2–9 are protein-coding, while exon 1 consists of the 5’-untranslated region harboring multiple promoter and transcription start sites. The alternative splicing of hGRs generates different isoforms, with hGRα and hGRβ as the best characterized ones so far. While hGRα binds to GCs and translocates from the cytoplasm to the cell nucleus to exert transcriptional effects, hGRβ is mostly localized in the nucleus and exerts as a negative inhibitor of hGRα [36,37].

Classically, several common genetic polymorphisms, most commonly single nucleotide polymorphisms (SNPs), have been identified within the *NR3C1* gene, which results in a modified transcript that could have a varying impact in GC sensitivity. The main SNPs affecting function described to date in *NR3C1* are *Bcl*I (rs41423247), N363S (rs56149945), ER22/23EK (rs6189 and rs6190), and A3669G (rs6198) [38]. The *Bcl*I polymorphism involves a cytosine to guanine substitution downstream of exon 2 of the gene and has been related to hypersensitivity to GC treatment, as carriers of the G-allele display an increased tissue GC sensitivity [39,40]. The N363S polymorphism is also located in exon 2 of the gene, predicting an aspartic acid to serine change at the amino acid position 363, which has been associated with increased GC sensitivity [35,37]. The ER22/23EK genetic polymorphism is located on exon 2 and consists of an arginine to lysine substitution at the amino acid position 23 and associated with a decrease in GC sensitivity [41,42]. Furthermore, the adenine to guanine substitution at the nucleotide 3669 in exon 9 makes hGRβ mRNA more stable, thus resulting in an increased expression of hGRβ, which further reduces hGRα-mediated transcriptional activity [37].

A pilot genetic study assessed the association of 14 polymorphisms in the GC pathway with treatment response in 107 dexamethasone-treated patients with severe COVID-19 [43]. When patients did not respond to treatment and required oxygen, non-invasive ventilation, or mechanical ventilation, it was switched to methylprednisolone. In this study, carriers of the rs33388 (*NR3C1*) C allele had lower odds for intensive care unit (ICU) admissions (*p* = 0.023) compared to non-carriers. Furthermore, longer duration of hospitalization was found in rs33388 A allele homozygotes compared to the other patients (*p* = 0.006). The rs33388 A allele is associated with an increased expression of the hGRγ isoform [44], which has lower stability for binding to GREs than the hGRα isoform and may explain the poor response to GCs among carriers of the A allele. On the other hand, homozygotes for the G allele at A3669G (rs6198) in *NR3C1* needed longer hospitalization (*p* = 0.001), longer oxygen supplementation (*p* = 0.001), and higher odds for switching to methylprednisolone treatment (*p* = 0.046). The increased hGRβ expression and stability resulting from rs6198 polymorphism [37] might underlie the GC resistance. Therefore, while the rs33388 polymorphism was associated with better treatment outcomes, rs6198 was associated with worse outcomes in response to GCs.

Another study was conducted on patients with inflammatory bowel disease (IBD) [45] in which a panel of 21 GC response-related genes was analyzed. A total of 139 European patients with IBD were included in the study (77 individuals with Crohn’s disease (CD) and 62 individuals with ulcerative colitis (UC)). Patients were classified as GC-sensitive, GC-dependent, or GC-resistant according to the occurrence of exacerbations or remission. The study showed that G allele carriers of the rs56149945 polymorphism at *NR3C1* were more resistant to GC treatment (*p* = 0.002).

### 3.2. Genomic Studies of Glucocorticoid Response

Early genetic studies in this field primarily focused on variations in the *NR3C1* gene due to its direct role in the GC response. However, the potential for SNPs in other genes to influence GC response has also been explored. Nicolaides et al. [46,47,48] aimed to identify more genetic variation associated with the differential GC response in 101 healthy adults, of whom 22 were classified as extreme with respect to GC sensitivity (11 were classified as the most GC sensitive and other 11 as the most GC resistant). Their targeted sequencing analyses of the protein-coding and intron–exon junctions of the gene revealed that none of the 22 individuals carried any genetic difference in the *NR3C1* gene that could account for the varying responsiveness, reinforcing the idea that polymorphisms in other genes not directly related to GR encoding may also influence GC response. In this sense, several other genetic loci have been associated with the GC response to date (Table 1).

#### 3.2.1. Genomic Studies of Glucocorticoid Response in Asthma

Tantisira et al. [49] conducted a study to determine changes in lung function in response to inhaled corticosteroids (ICS) in 181 child–parent trios from the Childhood Asthma Management Program (CAMP) and found a functional *GLCCI1* variant associated with a poorer response. Functional analyses revealed that the identified SNP rs37972 of this study is in complete linkage disequilibrium (LD) with SNP rs37973, both associated with reduced *GLCCI1* expression. *GLCCI1* may serve as an early marker of GC-induced apoptosis [50], which is a key mechanism by which GCs alleviate lymphocytic and eosinophilic inflammation in asthma [51]. Consequently, the lower *GLCCI1* expression due to the genetic variation might reduce the apoptosis of inflammatory cells, thus leading to a weaker clinical response. Specifically, the patients homozygous for the rs37973 G allele have shown a significantly lower expression of *GLCCI1*, while homozygotes for the A allele associate with an increased expression of the gene. Altogether, rs37973 GG carriers appear to have a poorer response to ICS. Hu et al. [52] also correlated *GLCCI1* variation with asthma susceptibility and ICS response in the Han Chinese adult population. Furthermore, in a study based on a Japanese asthma cohort, Izuhara et al. [53] also found that variation at the *GLCCI1* gene correlated with pulmonary function decline in patients with asthma receiving long-term ICS treatment. More recently, Edris et al. [54] investigated the pharmacogenetic effects of 10 common genetic variants selected from the literature (including rs37973) in 597 adult patients with asthma from the Dutch population-based Rotterdam Study in the discovery stage and in 9842 patients from GERA cohort for the replication. They also found a poorer response to ICS among rs37973 G allele carriers. In this study, Edris and colleagues also found three other *TBXT* genetic variants (rs1134481, rs2305089, and rs3099266) associated with the ICS response. In this case, patients homozygous for the haplotype for these three SNPs (TCT) were associated with a better response to ICS, i.e., a decreased risk of recurrent exacerbations while using ICS.

Park et al. [55] performed a genome-wide association study (GWAS) in a Korean asthma cohort (*n* = 189) to identify genetic variants associated with a change in the percentage of forced expiratory volume in 1 s (FEV_1_) after ICS treatment. The variant rs11123610, which maps to the *ALLC* gene, reached the lowest *p*-value (*p* = 3.57 × 10^−7^). In silico analyses revealed correlation between the *ALLC* function and three variants (rs13418767, rs6754459, and rs13409104) in high LD with rs11123610. ALLC is a type of uricolytic enzyme that has lost its activity during vertebrate evolution. However, animal studies suggest that, instead of being entirely non-functional, the gene has low expression levels and a reduced substrate affinity [56]. Nevertheless, while it is not clear whether the ALLC enzyme has lost all its functionality in humans or not, chromosome 2, where the *ALLC* gene is located, contains several genes that have been associated with immunoglobulin E (IgE) levels [57] and FEV_1_ [58]. Further studies on the functionality of this enzyme in humans would provide a better understanding of its role in the differential response to CSs.

Another gene that has been related to ICS response is *CRHR1*. It encodes a receptor that binds to the corticotropin-releasing hormone, a regulator of the HPA pathway [59]. Several variants in the gene have been investigated, and two of them have been successfully replicated (rs1876828 and rs242941). Tantisira et al. [60] performed a genetic association study with longitudinal change in lung function in three asthma populations (one used as the discovery and two others for replication) and found that variants of *CRHR1* were associated with enhanced response to ICS in all three populations. Specifically, homozygous patients for the A allele in rs1876828 had a greater improvement in their FEV_1_ compared to G allele homozygotes. However, studies on rs242941 have yielded contradictory results. In a study performed by Tansitira et al. [60], carriers of the rs242941 TT genotype had a greater improvement in lung function compared with the CC genotype.

Conversely, other studies have found that patients homozygous for the rs242941 T allele have decreased FEV_1_ [61], increased risk of exacerbations [54], and poor lung function [62] while using ICS. Inconsistencies in these findings could be due to a different definition of outcome and the difficulty that controlling for modifiers entails (e.g., comorbidities and the use of other drugs).

*FCER2* encodes the low-affinity IgE receptor (CD23), which is involved in the downregulation of IgE. Rogers et al. [62] found that the minor allele of rs28364072 (C) in *FCER2* was associated with poor lung function response and recurrent exacerbations despite ICS use in children with asthma from the CAMP study. A replication of these findings was performed by Koster et al. [63] in 386 children from the PAC-MAN study and 939 children and young adults from the BREATHE cohort. They found the rs28364072 C allele to be associated with an increased risk of severe exacerbations, asthma symptoms, and poorer response to ICS, replicating the results obtained by Rogers and colleagues [62].

*FBXL7* gene has also been related to the ICS response. In vitro studies have shown that FBXL7 induces cell apoptosis, and, therefore, increased gene expression can induce cell and tissue injury [64]. Park et al. [65] aimed to identify novel genetic markers predicting a symptomatic response to ICS. In that study, a GWAS was performed in 124 white children from CAMP using scores from diary cards, and the top 100 ranked genetic variants were pursued for replication in 77 child and 220 adult patients. Analyses revealed one variant, rs10044254 (combined *p* = 9.16 × 10^−8^), associated with the decreased expression of *FBXL7* and improved symptomatic response to ICS in the two independent pediatric cohorts, but not in the adult patients. Meanwhile, carriers of two copies of the reference allele (A) showed decreased symptom scores, and the patients homozygous for the variant allele (G) had poorer symptom responses.

*CMTR1* is a protein-coding gene involved in mRNA capping [66]. Dahlin et al. [67] conducted a GWAS of asthma exacerbations in 806 European patients with asthma from two population-based biobanks (BioVu and PMRP) and found that two SNPs (rs2395672 and rs279728) in *CMTR1* were associated with an increased risk of exacerbations in both populations, while rs4271056 was associated with decreased risk. The mRNA transcript of the gene showed a significant differential expression in nasal lavage samples from patients with asthma during acute exacerbations, which may indicate a role of *CMTR1* in the development of this condition.

In 2020, Dahlin and colleagues [68] performed a genome-wide interaction study (GWIS) of genetic variation and age on ICS response (defined by exacerbations) in a total of 1321 adult and child patients with asthma of European ancestry from five independent cohorts. A total of 107 genome-wide suggestive (*p* < 10^−5^) age-by-genotype interactions were detected, two of which met genome-wide significance (*p* < 10^−8^) (rs34631960 in *THSD4* and rs2328386 in *HIVEP2*). According to these results, rs34631960 was associated with a 2-fold increase in exacerbation risk on ICS with age, while rs2328386 was associated with approximately a 0.3–0.5-fold decrease in risk.

Wang et al. [69] conducted a GWAS of ICS response in older European adults with asthma from three different cohorts. The ICS response was measured by the absence of oral CS (OCS) bursts and the absence of asthma exacerbations according to diagnosis codes. Four variants (rs138717703, rs77506063, rs116023293, and rs145325916) near the *PTCHD4* gene met genome-wide significance on meta-analysis for the OCS burst outcome in 5710 subjects from GERA, 676 subjects from MGB Biobank, and 465 subjects from the Rotterdam Study. In the meta-analysis for the asthma-related exacerbations outcome, 4541 subjects from GERA and 505 subjects from MGB Biobank were included (the Rotterdam Study did not have data on asthma-related exacerbations), and none of the variants met genome-wide significance. Yet, 152 SNPs with suggestive statistical significance (*p* < 5 × 10^−5^) were validated across both cohorts for this outcome. SNPs associated with ICS response might vary depending on the outcome measured, and differences in how the two outcomes were derived in this study may have contributed to the absence of overlap in the top SNPs.

Although many studies have investigated the genetic component of ICS response in patients of European and Asian ancestry, Hernandez-Pacheco et al. [70] aimed to identify genetic variants associated with asthma exacerbations in ICS-treated admixed children and young adults (Hispanics/Latinos and African Americans) for the first time. In the discovery phase, a GWAS performed in 1347 admixed children prioritized 15 variants, which were selected for replication in 1697 European subjects. Located within the intergenic region of *APOBEC3B* and *APOBEC3C*, rs5995653 showed evidence of nominal replication (*p* = 7.52 × 10^−3^*)*. The polymorphism was also evaluated for association with change in FEV_1_ after 6 weeks of ICS treatment in 166 ICS users from the SLOVENIA study (the only cohort included in the analyses that had FEV_1_ data) and was nominally associated with an improvement of FEV_1_ levels (*p* = 4.91 × 10^−3^).

Some GWASs have also been conducted to identify genetic variants associated with side effects of GC therapy. In this regard, Park and colleagues [71] aimed to identify genetic predictors of OCS-induced adverse effects on bone mineral accretion (BMA) in children with asthma, as OCSs have been previously related to adverse effects on bone mineral density [72,73]. To that end, a GWAS of OCS-induced BMA in 489 white children from the CAMP study was performed. Two variants (rs9896933 and rs2074439) were identified and associated with decreased BMA and related to the tubulin ϒ pathway. The variant rs9896933 is located on *TBCD* gene and reached genome-wide significance (*p* = 3.15 × 10^−8^) when associated with decreased BMA. The top-ranked 2000 SNPs in that GWAS were selected, and whether these SNPs also had cis-regulatory effects on dexamethasone-induced gene expression in osteoblasts were then determined. The variant rs2074439 (*p* = 8.64 × 10^−4^) mapped to *TUBG1* and showed strong cis-regulatory effects on dexamethasone-induced tubulin ϒ gene expression in osteoblasts. Furthermore, Park and colleagues found that BMA worsened with an increased dose of prednisone as the copies of the risk alleles at the two variants increased.

HPA axis suppression is one of the most serious adverse effects of CS therapy. For that reason, Hawcutt and colleagues [74] aimed to identify genetic variants associated with susceptibility to CS-induced HPA suppression (defined as peak cortisol less than 350 nmol/L in children and less than 500 nmol/L in adults). A total of 499 children with asthma treated with ICS were enrolled for the discovery stage, 81 for the pediatric validation cohort, and 78 adults with COPD for the adult validation cohort. A variant intronic to *PDGFD* (rs591118) was prioritized in the discovery cohort (*p* = 5.8 × 10^−8^) and was successfully replicated in both validation cohorts.

#### 3.2.2. Genomic Studies of Glucocorticoid Response in Acute Lymphoblastic Leukemia

Whilst many studies on ICS response in asthma have been published, much fewer genetic association studies have been conducted in other conditions. Some studies have explored the effects of GC therapy in acute lymphoblastic leukemia (ALL) patients, as GCs are widely used for ALL treatment, but one of their major adverse effects in these patients is osteonecrosis [75,76]. Karol and colleagues [77] explored the genetics of GC-associated osteonecrosis in patients with ALL. A total of 2285 children with ALL were included in the discovery phase, where the variant rs10989692 (in *GRIN3A*) was associated with osteonecrosis (*p* = 3.59 × 10^−7^). The association was later validated in two replication cohorts including 361 ALL child patients and 309 non-ALL patients (combined *p* = 2.68 × 10^−8^).

Ramsey et al. [78] carried out a GWAS by analyzing 14 pleiotropic GC phenotypes (including undesired side effects) in 391 patients with ALL treated with dexamethasone. The analyses identified two SNPs (rs2243057 and rs6453253) located in the *F2RL1* gene, which plays a role in hemostasis, thrombosis, and inflammation.

Shinohara and colleagues [79] performed a GWAS in 72 leukemic cell lines from Japanese patients with ALL aiming to identify genetic variants associated with prednisolone and dexamethasone sensitivity and with *NR3C1* gene expression. IC50 values (defined as the concentration required to kill 50% of the in vitro cells) of prednisolone associated with the variant rs904419, an intergenic SNP to *FRMD4B* and *MITF* (*p* = 4.34 × 10^−8^). Regarding dexamethasone sensitivity, a suggestive association (*p* = 1.43 × 10^−6^) with rs2306888 (in *TGFBR3*) was put forward. Finally, the variant rs11982167 located in *PLEKHA8* showed a suggestive association with the *NR3C1* gene expression (*p* = 6.44 × 10^−8^), which may affect GC responsiveness, as it encodes the GR. Although these variants have not been identified in a cohort-based study, in vitro identification of genetic variants related to GC sensitivity could also provide insight into potential drug targets.

#### 3.2.3. Genomic Studies of Glucocorticoid Response in Other Acute and Chronic Diseases

Interestingly, one study addressed the glucocorticoid response and outcome in ARDS and COVID-19 patients [80]. Jalkanen and colleagues found one SNP (rs9984273) of the interferon α/β receptor subunit 2 (*IFNAR2*) gene, corresponding to a binding motif for the GR, associated with the GC response. They found that carriers of the T allele of the SNP have a poor outcome when treated with IFN β along with GCs. Meanwhile, the minor allele C and glucocorticoid use were associated with higher 28-day patient survival compared to T allele carriers. Overall, the study suggested that co-treatment with IFN β and GC should be avoided in homozygous rs9984273 T allele patients.

Aiming to identify the genetic determinants of long-term effect FEV_1_ decline related to ICS therapy in patients with COPD, a genotype-by-ICS (triamcinolone versus placebo) interaction GWAS was performed on 802 participants from the LHS-2 cohort for the discovery GWAS and 199 from the ABC cohort for the replication stage [81]. No loci were found to meet genome-wide significance. However, five variants met the secondary criteria at a *p*-value cut-off of 5 × 10^−6^. From these loci, rs111720447 on chromosome 7 showed statistically significant replication with the same effect direction (6.0 × 10^−5^). Although rs111720447 did not reach genome-wide significance, ENCODE data suggest that it is located near a GR binding site in the alveolar cell line A549 after treatment with dexamethasone, which further supports the genetic association.

In a study performed by Štampar and colleagues in COVID-19 patients [43], carriers of the rs35599367 (*CYP3A4*) T allele had higher odds for ICU admission (*p* = 0.01). Longer hospitalization was observed in carriers of the rs35599367 T allele compared to the rest of patients (*p* = 0.02). Finally, the duration of oxygen therapy was also longer in rs35599367 TC genotype carriers than in the CC genotype carriers (*p* = 0.04).

In the above-mentioned study performed in IBD patients [45], the G allele of rs2817033 at the *FKBP5* gene and a deletion 306-7delT of rs61763106 at the *MAPK14* gene were associated among patients with UC with GC sensitivity (*p* = 0.040) and with dependency and resistance to GC (*p* = 0.041), respectively. Among CD patients, the C allele of rs2032583 at the *ABCB1* gene was associated with GC resistance (*p* = 0.034).

Although many genomic studies have been conducted to identify biomarkers of CS response in asthma and, to a lesser extent, ALL, few have addressed GC response in other diseases such as COVID-19, COPD, sepsis, or ARDS, among others. However, some studies have found that CS treatment has been associated with delayed viral clearance and increased mortality risk in COVID-19 patients [82,83,84], and with increased pulmonary bacterial load and mucus production in COPD virus-induced exacerbations [85]. Therefore, the inter-individual variability of CS response among patients poses a challenge to treatment outcomes. Further studies focusing on these diseases to determine which patients may not benefit from CS treatment could enhance clinical management and lead to better outcomes.

In summary, pharmacogenomic studies play a significant role in unraveling genes related to interindividual differential GC responses. A few studies have identified to date common genetic variants that are associated with responsiveness to treatment or susceptibility to adverse effects (Table 1). However, despite the efforts that have been made in this area, there are still no variants described with a high level of evidence in PharmaGKB (https://www.pharmgkb.org; accessed on 15 July 2024), which further highlights the need for studies that provide solid evidence. Pharmacogenomic studies have unique challenges, including the difficulty that ensuring well-defined phenotypes across studies, drug response endpoints, studies in larger sample sizes, the adequate representation of genetic diversity of populations, and controlling for confounding factors (e.g., polypharmacy and drug–drug interactions, comorbidities, GC dose and administration route) entails. To improve the robustness of results in future genetic association studies in this field, it is necessary to increase the sample size of studies and including patients with a well-defined phenotype while controlling for confounding factors.

**Table 1:** Summary of key genes associated with glucocorticoid response derived from genomic studies.

Study	Sample	Condition(s)	GC	Outcome(s)	SNP(s)	Gene(s)	*p*-Value(s) *
[43]	107 hospitalized patients	COVID-19	Dexamethasone	ICU admission, duration of hospitalization	rs33388	*NR3C1*	0.023, 0.006
[43]	107 hospitalized patients	COVID-19	Dexamethasone	Duration of hospitalization, duration of oxygen therapy, switch to methylprednisolone	rs6198	*NR3C1*	0.001, 0.001, 0.046
[45]	62 patients with UC	IBD	Methylprednisolone, hydrocortisone or budesonide	GC response (exacerbations or remission)	rs56149945	*NR3C1*	0.002
[49]	118 child–parent trios	Asthma	Budesonide	Changes in FEV_1_	rs37972	*GLCCI1*	<0.05
[52]	182 patients and 180 healthy controls	Asthma	Fluticasone propionate	Changes in FEV_1_	rs37972, rs37973 and rs11976862	*GLCCI1*	<0.05
[53]	224 patients	Asthma	ICS (not specified)	Changes in FEV_1_	rs37973	*GLCCI1*	<0.05
[54]	597 patients in the discovery and 9842 in the replication	Asthma	ICS (not specified)	Asthma exacerbations	rs37973	*GLCCI1*	<0.005
[54]	597 patients in the discovery and 9842 in the replication	Asthma	ICS (not specified)	Asthma exacerbations	rs1134481, rs2305089 and rs3099266	*TBXT*	<0.005
[55]	189 patients	Asthma	Fluticasone propionate	Changes in FEV_1_	rs11123610	*ALLC*	3.57 × 10^−7^
[60]	470 patients in the discovery and 647 in the replications	Asthma	Flunisolide, budesonide, and triamcinolone	Changes in FEV_1_	rs1876828 and rs242941	*CRHR1*	<0.05
[61]	189 patients	Asthma	Fluticasone	Changes in FEV_1_	rs242941	*CRHR1*	2.07 × 10^−3^
[54]	597 patients in the discovery and 9842 in the replication	Asthma	ICS (not specified)	Asthma exacerbations	rs242941	*CRHR1*	<0.005
[62]	311 child patients	Asthma	Budesonide	Changes in FEV_1_	rs242941	*CRHR1*	0.05
[62]	311 child patients	Asthma	Budesonide	Changes in FEV_1_	rs28364072	*FCER2*	0.006
[63]	1325 child patients	Asthma	ICS (not specified)	Asthma exacerbations	rs28364072	*FCER2*	0.0004
[65]	124 child patients in the discovery and 77 in the replication	Asthma	Budesonide and fluticasone	Asthma symptoms **	rs10044254	*FBXL7*	9.16 × 10^−8^
[67]	806 patients	Asthma	Beclomethasone, budesonide, ciclesonide, flunisolide, mometasone, or triamcinolone	Asthma exacerbations	rs2395672, rs279728, and rs4271056	*CMTR1*	2.32 × 10^−6^, 2.64 × 10^−6^, and 2.77 × 10^−6^
[68]	1321 adult and child patients	Asthma	ICS (not specified)	Asthma exacerbations	rs34631960	*THSD4*	3.64 × 10^−8^
[68]	1321 adult and child patients	Asthma	ICS (not specified)	Asthma exacerbations	rs2328386	*HIVEP2*	4.98 × 10^−8^
[69]	6851 older adult patients	Asthma	ICS (not specified)	OCS bursts	rs138717703, rs77506063, rs116023293, and rs145325916	*PTCHD4*	3.09 × 10^−9^, 3.09 × 10^−9^, 7.65 × 10^−9^, and 8.99 × 10^−9^
[70]	1347 child patients in the discovery and 1697 patients in the replication	Asthma	ICS (not specified)	Asthma exacerbations	rs5995653	Intergenic *APOBEC3B/APOBEC3C*	7.52 × 10^−3^
[70]	166 patients	Asthma	ICS (not specified)	Changes in FEV_1_	rs5995653	Intergenic *APOBEC3B/APOBEC3C*	4.91 × 10^−3^
[71]	489 child patients	Asthma	Prednisone	BMA	rs9896933 and rs2074439	*TBCD* and *TUBG1*	3.15 × 10^−8^ and 2.74 × 10^−4^
[74]	499 child patients in the discovery, 81 in the child validation, and 78 adults with COPD in the adult validation cohort	Asthma/COPD	ICS (not specified)	HPA suppression	rs591118	*PDGFD*	3.5 × 10^−10^
[77]	2285 child patients in the discovery, 361 ALL child patients, and 309 non-ALL patients in the validation	ALL	Dexamethasone or prednisone	Osteonecrosis	rs10989692	*GRIN3A*	2.68 × 10^−8^
[78]	391 child patients	ALL	Dexamethasone	14 pleiotropic glucocorticoid phenotypes	rs2243057 and rs6453253	*F2RL1*	2.68 × 10^−5^ and 2.77 × 10^−4^
[79]	72 leukemic cell lines	ALL	Prednisolone	IC50	rs904419	Intergenic *FRMD4B*/*MITF*	4.34 × 10^−8^
[79]	72 leukemic cell lines	ALL	Dexamethasone	IC50	rs2306888	*TGFBR3*	1.43 × 10^−6^
[79]	72 leukemic cell lines	ALL	-	*NR3C1* gene expression	rs11982167	*PLEKHA8*	6.44 × 10^−8^
[80]	296 adult patients	ARDS/COVID-19	Not specified GC with IFN β-1a	28-day mortality	rs9984273	*IFNAR2*	<0.001
[81]	802 patients in the discovery and 199 in the replication	COPD	Triamcinolone or fluticasone	Changes in FEV_1_	rs111720447	-	discovery = 4.8 × 10^−6^, replication = 5.9 × 10^−5^
[43]	107 hospitalized patients	COVID-19	Dexamethasone	ICU admission, duration of hospitalization, duration of oxygen therapy	rs35599367	*CYP3A4*	0.01, 0.02, 0.04
[45]	62 patients with UC	IBD	Methylprednisolone, hydrocortisone, or budesonide	GC response (exacerbations or remission)	rs2817033	*FKBP5*	0.04
[45]	62 patients with UC	IBD	Methylprednisolone, hydrocortisone, or budesonide	GC response (exacerbations or remission)	rs61763106	*MAPK14*	0.041
[45]	77 patients with CD	IBD	Methylprednisolone, hydrocortisone, or budesonide	GC response (exacerbations or remission)	rs2032583	*ABCB1*	0.034

COVID-19: coronavirus disease 2019; UC: ulcerative colitis; IBD: inflammatory bowel disease; GC: glucocorticoid; SNP: single nucleotide polymorphism; ICU: intensive care unit; FEV_1_: forced expiratory volume in 1 s; ICS: inhaled corticosteroid; OCS: oral corticosteroid; BMA: bone mineral accretion; COPD: chronic obstructive pulmonary disease; HPA: hypothalamic–pituitary–adrenal axis; ALL: acute lymphoblastic leukemia; IC50: concentration required to kill 50% of the in vitro cells; ARDS: acute respiratory distress syndrome; and CD: Crohn’s disease. * In studies with validation cohorts, the *p*-value given corresponds to the meta-analysis across the cohorts included. ** Derived scores from daybook cards.

## 4. Transcriptomics and Epigenetics of Glucocorticoid Response

Transcriptomic analyses allow the assessment of gene expression at a specific time and in a specific context, enabling the direct observation of which genes are activated or repressed following GC administration, thus providing insights into mechanisms of action. Furthermore, these studies allow for the comparison of transcriptomic profiles between responders and non-responders to identify specific genes or pathways that may be differentially regulated. Epigenetics adds another layer of information by revealing how gene expression is regulated through reversible changes, such as DNA methylation or histone modifications. These two approaches, separate or in combination, can help to identify biomarkers or gene signatures that could predict CS efficacy, enabling more personalized treatment approaches and helping to avoid ineffective therapies in non-responders.

Davenport and colleagues [86] defined two distinct sepsis response signatures (SRSs) in the transcriptomic profile of adult patients with sepsis (270 patients in the discovery cohort and 114 in the validation cohort), SRS1 and SRS2, based on a set of seven predictive genes of group membership (*DYRK2*, *CCNB1IP1*, *TDRD9*, *ZAP70*, *ARL14EP*, *MDC1*, and *ADGRE3*) assayed on peripheral blood leukocytes. SRS1 was significantly associated with reduced survival and a more severe illness. These SRS endotypes were later studied by Antcliffe et al. [87] to determine if they influence the differential response to CSs in septic shock patients. Samples were available from 176 patients: 82 for the SRS1 endotype, and 94 for SRS2, and the study revealed that patients with the SRS2 endotype who were treated with hydrocortisone had increased mortality compared to those on placebo.

In another study, Baines and colleagues [88] conducted a gene expression study in sputum samples to identify a gene signature that could discriminate asthma inflammatory phenotypes. A signature of six genes (*CLC*, *CPA3*, *IL1B*, *DNASE1L3*, *ALPL*, and *CXCR2*) allowed for distinguishing eosinophilic asthma from other phenotypes in the cohort. Furthermore, the gene signature predicted the response to inhaled fluticasone. In patients with eosinophilic asthma, the ICS treatment reduced the expression of *CLC*, *CPA3*, and *DNASE1L3*. The same six-gene signature was tested to determine whether it could also predict the response to oral prednisolone treatment [89]. The study showed that the gene signature was a better predictor of the OCS response than eosinophil measures in blood and sputum. Prednisolone treatment reduced *CLC* and *CPA3* expression, while *DNASE1L3*, *IL1B*, *ALPL*, and *CXCR2* expression remained unaltered.

Some COPD patients do not benefit from continuing with ICS treatment, and determining which patients may safely sustain ICS withdrawal would improve treatment decisions. Ditz and colleagues [90] demonstrated that the sputum transcriptome of COPD patients predicts exacerbations after the withdrawal of ICS better than the eosinophil levels. A gene expression signature (including *LGALS12*, *ALOX15*, *CLC*, *IL1RL1*, *CD24*, and *EMR4P*) was associated with shorter time to the first exacerbation after ICS withdrawal and demonstrated a higher predictive value than sputum eosinophil levels among COPD patients. Furthermore, a higher baseline expression of three genes (*SEPP1*, *CCDC152*, and *ALG3*) was associated with a delayed onset of the first exacerbation after ICS withdrawal.

Kuo et al. [91] aimed to determine molecular endotypes of asthma by analyzing sputum cell transcriptomics from 104 asthma patients. Out of the three transcriptome-associated clusters (TACs) obtained by hierarchical analysis, TAC1 defined severe asthma patients with acute exacerbations, nasal polyps, airflow obstruction, and high OCS dependency. The gene signature defined by TAC1 included a total of 20 genes (*IL1R1*, *PRSS33*, *CLC*, *GPR42*, *LGALS12*, *SOCS2*, *ALOX15*, *TARP*, *ATP2A3*, *TRGV9*, *FAM101B*, *CD24*, *CRCLF2*, *TRGC2*, *TPSB2*, *OLIG2*, *HRH4*, *CPA3*, *CCR3*, and *VSTM1*). An independent study then assessed whether these asthma TACs could be used to classify patients with COPD and if they were associated with steroid sensitivity [92]. In that study, TAC2 and TAC3 signatures were unaffected by ICS therapy. However, the TAC1 signature decreased with CS treatment, suggesting that this endotype was steroid-sensitive. In fact, after ICS withdrawal, the signature reversed. This is consistent with Ditz et al.’s findings [90], where the gene signature found in COPD patients included several genes from the TAC1 endotype (*LGALS12*, *ALOX15*, *CLC*, *IL1RL1*, and *CD24*) and was associated with shorter time to the first exacerbation after ICS withdrawal.

Another study focused on CS responsiveness in a small cohort of 16 patients with chronic rhinosinusitis with nasal polyps [93]. Nasal polyp biopsies were obtained before and after 14-day treatment with oral methylprednisolone, and patients were classified as responders or non-responders. The results revealed higher expression of the type 2 inflammatory response genes (*CCL13*, *IGHE*, *CCL18*, *CCL23*, *CCR3*, and *CLC*) and lower levels of *LACRT*, *PPDPFL*, *DES*, *C6*, *MUC5B*, and *SCGB3A1* associated with better response to systemic GCs.

With the aim to identify biomarkers of epithelial cell dysfunction and the effects of CS treatment on asthma patients, Woodruff and colleagues [94] collected cells by bronchoscopy and examined the transcriptomes with genome-wide gene expression microarrays. They found that, while high baseline gene expressions of *CLCA1*, *POSTN*, and *SERPINB2* are associated with a good clinical response to fluticasone, high expressions of *FKBP51*, which modulates the GR activity [95], are associated with a poor response.

In a study of GC resistance in ALL patients, leukemia cells from 173 children were tested in vitro for sensitivity to prednisolone [96]. The study found an overexpression of the apoptotic gene *MCL1* and the downregulation of several transcription-associated genes (e.g., *SMARCB1*, *PRPF18*, and *CTCF*) in the prednisolone-resistant ALL cells.

Epigenetic changes causing the overexpression of *CASP1* and *NLRP3* genes have shown to decrease GC sensitivity in leukemic cells [97]. A study of prednisolone and dexamethasone sensitivity of primary leukemic cells from 444 newly diagnosed ALL patients was conducted by Paugh and colleagues [97]. Gene expression, DNA methylation, and ChIP-seq analyses showed a higher expression of *CASP1* and its activator *NLRP3* as a result of lower somatic methylation of these gene promoters in GC-resistant leukemic cells. The overexpression of these genes induced GC resistance through cleavage of the GR by *CASP1*, thereby reducing functional GR levels and the transcriptional effects of GCs.

In the CAMP trial, another study examined the association between peripheral blood DNA methylation and budesonide response in 152 asthma children [98]. The response to ICS was measured as changes in FEV_1_. The relative hypermethylation of five CpG sites (cg20434811, cg02822723, cg14066280, cg27254601, and cg23913400) and relative hypomethylation of two others (cg24937126 and cg24711626) were associated with improvement in FEV_1_. Notably, the relative hypermethylation of cg27254601 was associated with both an increase in FEV_1_ and *BOLA2* gene expression.

In another study conducted by Wang and colleagues [99], epigenome-wide DNA methylation was correlated with gene expression in peripheral blood from 394 ICS-treated asthma subjects from three independent and ethnically diverse cohorts (CAMP, BAMSE, and GACRS). Treatment response was defined as the absence of emergency department (ED) visits and the absence of OCS bursts. The results showed that relative hypomethylation of the CpG site cg00066816 was associated with the absence of ED visits in the study cohorts and with a lower *IL12B* expression in BAMSE. Furthermore, relative hypermethylation of the CpG site cg04256470 was associated with absence of OCS use in the CAMP and the GACRS and with *CORT* expression in the CAMP. Notably, *CORT* encodes the neuropeptide cortistatin, which regulates the HPA axis and exerts anti-inflammatory actions, thus suggesting that regulation of cortistatin by DNA methylation may modulate ICS sensitivity [100,101]. In conclusion, Wang and colleagues found that the differential DNA methylation of *IL12B* and *CORT* genes was associated with ICS response in persistent childhood asthma.

Overall, transcriptomic and epigenomic studies represent a promising approach for unraveling the molecular basis of differential CS response. However, larger studies addressing cohorts with more phenotypic homogeneity are needed in order to overcome the challenges that this type of studies implies.

## 5. Proteomics and Metabolomics of Glucocorticoid Response

Due to different factors, including the involvement of different regulatory mechanisms of transcription and translation, the targeted study of proteins and their post-translational modifications has enormous potential to further contribute to the discovery of biomarkers of drug response.

A study performed by Wang et al. [102] assumed that Seasonal Allergic Rhinitis (SAR) patients with high (HR) and low response (LR) to treatment might have different nasal fluid protein profiles and reasoned that they may act as potential biomarkers for GC treatment response. Nasal lavage samples from 40 SAR patients were collected, and the top 40 proteins discriminating HR and LR were extracted for detailed analyses. Several proteins differed before and after treatment, but specifically, ORM, FGA, and APOH proteins decreased significantly in HR but not in LR after treatment.

Jiang and colleagues [103] aimed to identify potential prognostic protein biomarkers in leukemia cell lines REH, 697, Sup-B15, and RS4; 11. After prednisolone treatment, cells were separated into resistant (REH) and sensitive (697, Sup-B15, and RS4; 11). After separation by 2D gel electrophoresis, a total of 870–1200 protein spots were detected. From those, 77 and 17 protein spots were identified as differentially expressed in sensitive and resistant (respectively) cell lines. The PCNA protein was downregulated after treatment in all three sensitive cell lines but remained unaltered in the resistant REH cell line. The protein expression level changes in PCNA were validated in paired bone marrow samples from ALL patients (thirty-five good responders and eight poor responders). The results showed that PCNA was significantly downregulated in good responder patients at day 8 after treatment but remained unaltered in poor responders. Altogether, PCNA levels were highly predictive of prednisolone response in ALL. Nicholson and colleagues [104] compared pre- and post-dexamethasone treatment, the protein profiles of the B-lineage ALL GC-sensitive cell line PreB 697, and its GC-resistant subline R3F9. Their results revealed that PAX5, a transcription factor involved in B-cell development, was differentially regulated in PreB 697 and R3F9 cell lines after treatment, as it was decreased in the resistant line. Furthermore, the basal protein level of PAX5 was also lower in R3F9 compared with the GC-sensitive PreB 697 cell line. This suggests that PAX5 is a transcriptional target of GCs and that its basal protein expression is associated with responsiveness to GC treatment. To further investigate the causes of the decreased PAX5 expression in R3F9, gene copy number variation (CNV) and a mutational screening of all coding exons were conducted in both cell lines. However, no evidence for changes in acquired mutations or in CNVs in the resistant line were found. The transcripts of *PAX5* were similar in all cell lines. Taken together, these results suggest that the reduced PAX5 protein level in GC-resistant lines may be due to post-transcriptional modifications.

Metabolomic studies could uncover metabolites that could serve as biomarkers of GC response and highlight the pathways underlying GC response, while also providing the early detection of severe side effects of GC use. The profiles of 214 metabolites from 20 healthy male plasma were recorded for 4 consecutive days in different time points before and after a single dose of 4 mg of dexamethasone to investigate the effects of GCs in the metabolome [105]. After midday at day 3, the volunteers received a single oral dose of GCs, and the results revealed that 150 of 214 metabolites were significantly dysregulated after the treatment. Also, dexamethasone almost completely suppressed and dysregulated the pre-treatment circadian rhythm. The observed changes included the following: the suppression of endogenous steroid production (that relates to the main side effect of GC use, that is, HPA suppression), altered catecholamine levels (linked to psychological side effects), changes in carbohydrate profiles (associated with steroid-induced diabetes), the modulation of polyunsaturated fatty acids (that is linked to increased risk of infection, atherosclerosis, hypertension, and diabetes), increased protein degradation (related to muscle atrophy), and high trans-4-hydroxyproline levels (which pose a risk for osteoporosis). Altogether, this study revealed that a single dose of dexamethasone resulted in several metabolome changes that relate to typical CS side effects. In another study, urine samples from responder and non-responder children with asthma treated with GCs were used to perform high-resolution metabolomics to identify possible biomarkers of GC resistance [106]. The metabolic phenotypes were analyzed, and levels of 30 metabolites related to tyrosine metabolism, the degradation of aromatic compounds, and glutathione metabolism were significantly different between responders and non-responders. Aiming to explore plasma metabolomic profiles associated with asthma exacerbations during ICS treatment, Kachroo and colleagues [107] conducted a study in 170 asthma patients from the Mass General Brigham Biobank. Of the seven-hundred eighty-three metabolites tested, eight showed a significant association with exacerbations. Four of these metabolites (cortisone, cortisol, tetradecanedioate, and hexadecanedioate) belong to the lipid super-pathway, and their levels were decreased in association with increased exacerbations, while the remaining four metabolites (mannitol/sorbitol, urea, 5-methylthioadenosine, and 1-carboxyethylvaline) belong to carbohydrate and amino acid super-pathways and were positively associated with exacerbations.

Taken together, these studies demonstrate that plasma metabolomic profiles of patients taking GCs may differ between responders and non-responders and prove the value that metabolomics can add to the study of GC resistance. However, despite the potential that metabolomic studies of GC resistance have, they are still scarce in the literature.

## 6. Conclusions and Future Prospects

CSs have been the treatment of choice for several conditions since their discovery in 1948 [10]. Due to their potent anti-inflammatory and immunosuppressive activity, their therapeutic effects could benefit a wide range of conditions such as asthma, ARDS, COPD, pneumonia, severe COVID-19, ALL, sepsis and severe infections, among others. However, their mechanisms of action usually lead to a broad side-effect profile [4,5], and, furthermore, some patients do not even respond to the treatment [6,7]. For that reason, their use is a matter of discussion, and clinical guidelines are continually being updated to align with the latest evidence on intake recommendations.

Several studies have been conducted to date, aiming to elucidate the genetic causes of the differential GC responses observed in clinical practice, which can lead to responsiveness biomarker discovery and, thus, tailored treatment therapies. Early studies primarily focused on assessing the variation in the *NR3C1* gene due to its direct role in GC response. However, the existing literature in this regard, while limited in number, supports that genetic variation in other genes might also be involved in the response. Some of the genes that are worth highlighting include *GLCCI1*, whose links with a poorer response to ICS in asthma patients have been replicated in different populations [49,52,53,54], and *FCER2*, with a variant associated with asthma exacerbations and poor lung function despite ICS use in two independent cohorts [62,63]. Conversely, for other genes such as the case of *CRHR1*, the evidence linking genetic variation with the GC response is still contradictory [54,60,61,62]. Differences in the response endpoint definition, ancestry or sample size, or the presence of other comorbidities and the use of other drugs may lead to conflicting results between studies. Findings should be interpreted with caution, considering the background of the cohorts involved (size, ancestry, phenotype, endpoint definition, age, comorbidities, etc.). There is no consensus on what constitutes a good or a bad response to GC treatment, and several variables have been used to determine response. It also depends on the disease that is in focus. Therefore, pharmacogenomic studies of GC response are challenged by the difficulty of ensuring the homogeneity of outcomes across studies and by the difficulty of identifying which outcome is the most appropriate to draw robust conclusions. Large-scale biomedical databases offer a unique opportunity to conduct such pharmacogenetic studies. However, it is quite common in those studies to have missing data for the outcome of interest in many cases. Therefore, increasing the representation of genetic diversity in the studies, selecting the subjects with a well-defined response endpoint, and controlling the confounders will improve the robustness of the results in the coming genetic association studies. Furthermore, although GWASs have identified several variants associated with GC response, these variants may have a small effect and limited predictive power. Previous studies have shown that evaluating the effects of multiple SNPs simultaneously can improve the predictive power of certain phenotypes [108]. In this sense, polygenic risk score (PRS) models can be used to infer genetic overlap between traits and predict phenotypes based on the genetic profile, besides providing an individual-level estimate of genetic liability for a trait [109]. Despite the promising applications of this approach in GC response studies, studies applying PRS methods are very limited and without interest in explaining the phenotypic variance of the endpoints under study. PRS may be useful to infer the genetic overlap of GC response between diseases and may be useful to detect association with GC response in small cohorts. However, for this to be a reality, the GWAS of pharmacogenetic responses that sustain PRS models face the above indicated limitations.

Integrating genome studies with other approaches such as transcriptomics and proteomics would add another layer of insight into the mechanisms by which GCs exert their effects and the pathways involved in their action and development of side effects. In fact, transcriptional gene signatures have been related to GC responsiveness, and they could define disease endotypes that behave differently under CS treatment. Proteomic and metabolomic studies have not been as extensively explored as other omics layers in this context yet. However, the few available studies certify that they constitute promising approaches for the discovery of biomarkers of GC response. Pharmacogenomic, transcriptomic, epigenomic, proteomic and metabolomic studies provide valuable insights of the GC response. However, these studies face important challenges such as the drug response endpoint definition and the difficulty in controlling for confounding factors (e.g., GC dose and the route of administration, comorbidities, polypharmacy, and drug–drug interactions). In addition, focusing on a single omics approach means that the upstream or downstream implications of the findings that they provide often remain unexplored. While single omics approaches show promising results, their broader implications are often underexplored, which translates into a need to move towards integrative approaches. Integrating different omics approaches could bridge the gap from genotype to phenotype and provide a more complete biological picture of the mechanisms involved in the differential GC response. Some studies in cancer have implemented integrative omics approaches, providing a better understanding and a clearer picture of the trait under study [110,111,112]. However, this approach has yet to be explored in the context of GC response.

Finally, even though GCs are extensively used in other acute conditions such as sepsis, severe COVID-19, or ARDS [113,114,115,116], most studies conducted so far to identify biomarkers of the GC response have focused on patients with asthma, with ALL, or in cell lines. Thus, an effort to address the genetic basis underlying the differential response to GCs and side-effect profiles in other patient studies, including critically ill patients, is necessary.

## Data Availability

This study did not generate new data.
